# Protozoan Parasites of *Sarcocystis* spp. in Rodents from Commercial Orchards

**DOI:** 10.3390/ani13132087

**Published:** 2023-06-23

**Authors:** Petras Prakas, Vitalijus Stirkė, Donatas Šneideris, Paulina Rakauskaitė, Dalius Butkauskas, Linas Balčiauskas

**Affiliations:** Nature Research Centre, Akademijos 2, 08412 Vilnius, Lithuania; vitalijus.stirke@gamtc.lt (V.S.); donatas.sneideris@gamtc.lt (D.Š.); rakauskaite.paulina@gmail.com (P.R.); dalius.butkauskas@gamtc.lt (D.B.); linas.balciauskas@gamtc.lt (L.B.)

**Keywords:** small mammals, orchards, Lithuania, *Sarcocystis*, infection rates, genetic identification, phylogeny

## Abstract

**Simple Summary:**

Small mammals not only play an important role in ecosystems, but they also can transmit a wide range of pathogens to humans and domestic animals. The data on protozoan *Sarcocystis* parasites in orchard-dwelling small mammals are still scarce. Members of the genus *Sarcocystis* form sarcocysts in the muscles of intermediate hosts and develop sporocysts in the intestines of definitive hosts. In the present study, 679 muscle samples of small mammals, collected in commercial orchards and berry plantations in Lithuania, were screened for *Sarcocystis* parasites via DNA analysis. The prevalence of *Sarcocystis* spp. was low as only nine pooled muscle samples were found to contain the parasites examined. Four species were identified in the examined small mammals, including two potentially new *Sarcocystis* species that were detected in the muscles of voles. The phylogenetic results suggested that birds and mammals are the definitive hosts of the *Sarcocystis* spp. identified in the current study.

**Abstract:**

Small mammals are an important group of wildlife that can transmit pathogens to humans and animals. There is a lack of comprehensive studies on the protozoan parasites of the genus *Sarcocystis* in agricultural areas. The aim of the current research was to evaluate the prevalence of *Sarcocystis* spp., and to identify the parasite species found in the skeletal muscles of rodents and insectivores from commercial orchards. A total of 679 muscle samples from small mammals, mainly rodents (n = 674), belonging to eight species were examined. Muscle samples were pooled into groups, then digested, and the presence of the *Sarcocystis* species was confirmed by molecular methods. The examined parasites were determined in five rodent species, *Apodemus agrarius*, *A*. *flavicollis*, *Clethrionomys glareolus*, *Microtus arvalis*, and *M*. *oeconomus*. The prevalence of *Sarcocystis* spp. was low: 2.23% in voles and 0.79% in mice. Based on a sequence comparison of *cox1* and *28S* rDNA, four species were identified: *S*. *myodes*, *Sarcocystis* cf. *strixi*, *Sarcocystis* sp. Rod1, and *Sarcocystis* sp. Rod2. This is the first report of *S*. *myodes* in *A*. *agrarius*, *A*. *flavicollis*, and *M. arvalis*. The identified species were most closely related to *Sarcocystis* spp., and were transmitted by predatory mammals and birds. Future studies are needed to describe the species morphologically, as well as to define the host spectrum and to evaluate their possible pathogenicity.

## 1. Introduction

Small mammals are a group of mammals distinguished by their relatively low body mass, short lifespan, and high fertility rate. This group includes more than 2500 species of rodents, 450 species of insectivores (eulipotyphlans), about 20 species of tree shrews (order Scandentia), but also other taxa that are not considered in this paper, such as marsupials [1,2]. Small mammals are important components of the food chain [3,4,5,6] for more than 75 species of predators in Northern and Central Europe [7,8,9]. They can transmit pathogens to humans, especially in residential areas [10,11,12], and to farm or domestic animals [13,14,15,16]. The main problems they cause are leishmaniasis, schistosomiasis, and *Leptospira*, as well as *Ricketsia* at lower latitudes.

According to long-term trapping data [17,18], four species of small mammals are commonly found in Lithuania with a proportion that is over 10% in their communities—the bank vole (*Clethrionomys glareolus*), the yellow-necked mouse (*Apodemus flavicollis*), the striped field mouse (*A. agrarius*), and the common shrew (*Sorex araneus*). Proportions of other four small mammal species, the common vole (*Microtus arvalis*), the root vole (*M. oeconomus*), the field vole (*M. agrestis*), and the pygmy shrew (*Sorex minutus*), accounted for 2–10% of all trapped individuals [18].

Ecological studies of the small mammals in commercial orchards in Lithuania have been carried out only in recent years [19,20,21]. However, the parasites of small mammals in these habitats have not been studied in Lithuania. Commercial orchards are anthropogenic habitats that are frequently visited by humans; thus, parasite surveys in this habitat are important for assessing the one-health risk [22]. Rodent-carried zoonotic protozoans are a threat to humans in cities [23] and agricultural areas [24]. Various protozoan pathogens were found in rodents from agricultural areas [25,26,27]; however, data on *Sarcocystis* in orchard-dwelling rodents are scarce.

The genus *Sarcocystis* encompasses globally distributed abundant protozoan parasites that are characterized by a two-host prey–predator life cycle. Sarcocysts are formed in the extra-intestinal tissues of intermediate hosts, mainly in muscles and the central nervous system, while the sporulation of oocysts occurs in the small intestine of definitive hosts [28,29,30,31]. Until now more than 200 *Sarcocystis* species have been described in mammals, birds, and reptiles [29,31]. Some species of *Sarcocystis* are pathogenic to their intermediate hosts, wildlife, and farm animals, as well as to humans [30].

Among the small mammals, the composition of the *Sarcocystis* species has been most comprehensively examined in rodents. More than 40 *Sarcocystis* are known to use rodents as their intermediate hosts [32]. However, the vast majority of these species were described and characterized using morphological methods [29]. Whereas approximately one-third of these species—*S*. *atheridis*, *S*. *dispersa* [33], *S*. *clethrionomyelaphis* [34], *S*. *cymruensis* [30,35], *S*. *glareoli*, *S*. *microti* [36,37], *S*. *muris* [37,38], *S*. *myodes* [32], *S*. *pantherophisi* [39,40], *S*. *ratti* [35,41], *S*. *singaporensis*, *S*. *zamani*, and *S*. *zuoi* [42,43,44,45,46,47,48]—have been examined with the help of DNA sequence analysis. Meanwhile, only two *Sarcocystis* species have been described in tree shrews, *S*. *scandentiborneensis* [31] and *S*. *tupaia* [49] and three valid species *S*. *attenuati* [50], *S*. *booliati*, and *S*. *russuli* [51,52] are known to infect eulipotyphlans.

Previous *Sarcocystis* parasite studies examining the muscles of small mammals from Lithuania and involving large numbers of animals (≥590) were carried out in wild nature [53,54,55]. *Sarcocystis* spp. were identified by the microscopical detection of sarcocysts in squashed and methylene-blue stained preparations. These studies demonstrated low *Sarcocystis* spp. infection rates, varying from 2.07% to 11.01%, and the parasites detected have not yet been characterized to the species level [53,54,55]. In contrast, molecular techniques can provide more detailed information on *Sarcocystis* species characterization and inter-species evolutionary relationships that cannot be determined by microscopy [56,57]. Therefore, the main objectives of this work were to identify members of the *Sarcocystis* species by molecular analysis and to determine the phylogenetic relationships of the species found in the skeletal muscles of small mammals collected in Lithuanian orchards. The *Sarcocystis* spp. diagnosis technique was based on a pooling of samples, muscle digestion, nested PCR, and a Sanger sequencing of the amplified fragments.

## 2. Materials and Methods

### 2.1. Sample Collection

**Figure 1:**
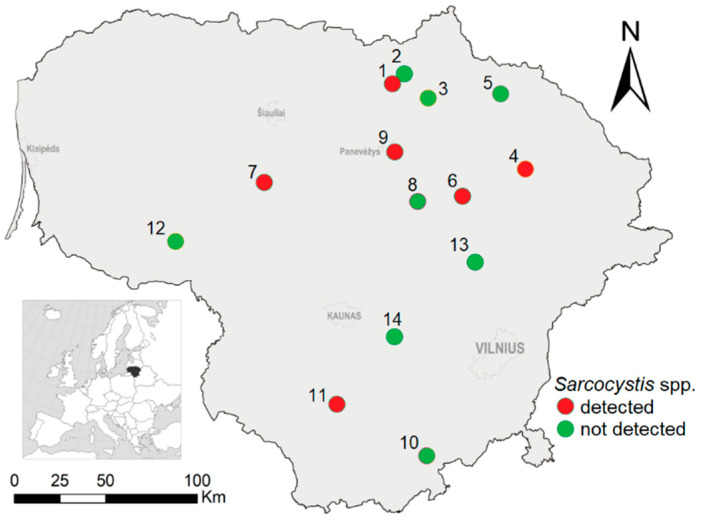
Investigation sites in Lithuania with a detection of *Sarcocystis* pathogens in the rodents indicated: 1—Aukštikalniai, 2—Naradava, 3—Mieliūnai, 4—Užpaliai, 5—Kalpokai, 6—Ažuožeriai, 7—Tytuvėnai, 8—Taujėnai, 9—Dembava, 10—Barčiai, 11—Luksnėnai, 12—Gaurė, 13—Šešuolėliai, and 14—Žiežmariai.

Small mammals were snap-trapped at 14 study sites, representing commercial orchards and berry plantations, across Lithuania in 2020 (Figure 1). We used the following standard trapping protocol [58]: in each sampling site, one to four lines with 25 traps at 5 m intervals were set, these were kept for three days and checked once a day in the morning. Bread soaked in sunflower oil was used as bait, and the bait was changed after rain or after it had been consumed by mammals, birds, insects, or slugs. In total, 679 small mammals belonging to eight species (*A. agrarius, A. flavicollis*, *C. glareolus*, *M. agrestis*, *M. arvalis*, *M. oeconomus*, *Sorex araneus*, and *S. minutus*) were trapped (Table 1). Skeletal muscle tissue from the individuals was used for the *Sarcocystis* infection study. All muscle tissues were frozen at −20 °C.

### 2.2. Sample Pooling and Muscle Digestion

Due to the vast number of samples, the collected animals were combined, by species and sites, into pools of 91. The number of individuals per pool varied between two and 10, with an average of 7.46 ± 0.25 animals per group. The average number per pooled sample was 8.11 ± 0.48 for *C*. *glareolus*, 7.81 ± 0.41 for *A*. *flavicollis*, 7.68 ± 0.54 for *A*. *agrarius*, 7.65 ± 0.41 for *M*. *arvalis*, 5 for *M*. *agrestis*, 3 for *S*. *minutus*, and 2 for both *M*. *oeconomus* and *S*. *araneus*.

The muscles of each pool were cut into small pieces and digested with pepsin, as described previously in [57]. The amount of muscle per pooled sample varied approximately between 1 and 50 g. Briefly, the chopped muscles were suspended in 15 mL of 0.9% saline solution, homogenized in a commercial blender at top speed for 2 min with breaks, incubated with a digestion solution at 37 °C for 1 h, and then centrifugated two–three times at 1600 rpm for 6 min. A total of 200 µL of sediments was used for the DNA extraction.

### 2.3. Molecular Examination

Genomic DNA from the digested muscle samples was extracted with the help of a PureLink Microbiome DNA Purification Kit (Invitrogen by Thermo Fisher Scientific, Waltham, MA, USA), which was utilized according to the manufacturer’s instructions.

Nested PCR and subsequent sequencing were used for the detection of *Sarcocystis* spp. in the examined pooled muscle samples. It was aimed to amplify fragments of four genetic loci, *18S* rDNA, *28S* rDNA, *cox1*, and *ITS1*. These loci were most commonly applied for the identification of *Sarcocystis* spp.; this was achieved by using small mammals as their intermediate hosts [41,50]. Primers were designed by a Primer 3 Plus program [59]. For the selection of primers, the numerous sequences of *Sarcocystis* spp. that were isolated from the small mammals were retrieved from GenBank and aligned by a CLC Sequence Viewer 8.0 (QIAGEN, Aarhus, Denmark). The aim was to design the primers to theoretically amplify as many as possible of the *Sarcocystis* species from small mammals. The list of primers used in the study is presented in Table 2.

The amplification of both steps of nested PCR was performed under the same conditions and via the same thermal protocol. PCRs were carried out in a 25 µL reaction volume containing 12.5 µL of DreamTaq PCR Master Mix (Thermo Fisher Scientific Baltics, Vilnius, Lithuania), 0.5 µM of each primer, 2 µL of template DNA, and 9.5 µL of nuclease-free water. The amplification started for 5 min at 95 °C, followed by 35 cycles of 45 s at 94 °C, 60 s at 52–60 °C (depending on the primer pair (Table 2)), 80 s at 72 °C, and ended with the final extension at 72 °C for 10 min. In each set of PCR positive and negative controls, water instead of template DNA were applied. During our previous investigations, the DNA extracted from the individual sarcocysts of *S*. *ratti* [41] and *S*. *myodes* [32] were used as positive controls. PCR products were visualized using 1.0% agarose gel electrophoresis.

The enzymatic purification of the amplified products was performed with alkaline phosphatase FastAP and exonuclease ExoI (Thermo Fisher Scientific Baltics, Vilnius, Lithuania). The amplification products were sequenced directly by using the forward and reverse second-step primers of the nested PCR. Sequencing was conducted using the Big-Dye^®^Terminator v3.1 Cycle Sequencing Kit (Thermo Fisher Scientific, Vilnius, Lithuania) and the 3500 Genetic Analyzer (Applied Biosystems, Foster City, CA, USA); both were utilized according to the manufacturer’s instructions. The chromatograms obtained were pure, without double or poly peaks.

The resulted sequences were compared with those of various *Sarcocystis* spp. with Nucleotide BLAST (http://blast.ncbi.nlm.nih.gov/, accessed on 17 January 2023). The genetic comparison of the obtained sequences was also made using the Heatmapper program [63]. Multiple alignments of *28S* rDNA and *cox1* sequences were obtained with the MUSCLE algorithm when implemented in MEGA7 [64]. The selection of the nucleotide evolution model best fitting dataset, as well as the generation of the phylogenetic tree under the Bayesian inference, was made on TOPALi v2.5 [65]. The resulted phylograms were visualized and edited in MEGA7. The final alignment that was generated employing *cox1* consisted of 619 nucleotide positions without any indels. Whereas the *28S* rDNA alignment was composed of 956 nucleotide positions with gaps. The JC + G and HKY + G evolutionary models were set for the *cox1* and *28S* rDNA analysis, respectively. For an evaluation of the robustness of the suggested phylogeny, a bootstrap test with 1000 replicates was performed. The 28S rDNA and *cox1* sequences of the *Sarcocystis* spp. that were isolated from the muscles of the small mammals obtained in the present study are available in GenBank (accession numbers OQ557453-OQ557461 and OQ558004-OQ558012, respectively).

### 2.4. Statistical Analysis

The prevalence estimates (in percent) and the 95% Cis for the small mammal species studied were calculated based on pooled samples [66,67]. We also calculated the prevalence and 95% CI for the investigation sites, as well as for the pooled samples of the voles, mice, and shrews (Table 3). The point estimation was conducted by employing the maximum likelihood method, maximizing the pooled likelihood function, and the CI was estimated by using a correction for skewness of the score function and the asymptotic confidence limits [68].

Differences in the prevalence of the identified *Sarcocystis* spp. were evaluated by conducting a Chi-squared test, which was calculated in WinPepi, ver. 11.39, and by using an exact Fisher’s P for the small and medium sample sizes [69]. Regarding the comparison of the prevalence of *Sarcocystis* spp. between the species and species groups (voles, mice, and shrews), the effect size was expressed according to an adjusted Cohen’s w [70].

## 3. Results

### 3.1. Prevalence of Sarcocystis *spp.* in Small Mammals

By molecular methods, *Sarcocystis* spp. were confirmed in nine pooled samples. Of the eight host species examined, *Sarcocystis* spp. were identified in five rodent species, i.e., in the four pooled samples of *M*. *arvalis*, in two samples of *A*. *flavicollis,* and in a single sample of *A*. *agrarius*, *C*. *glareolus*, and *M*. *oeconomus* (Table 3). The samples of the host species, which were negative for the screened parasites, were small and consisted of up to 10 individuals and one to two pooled groups. The overall prevalence of *Sarcocystis* spp. accounted for 1.38% (95% CI = 0.68–2.52). It should be noted that the prevalence of the *Sarcocystis* spp. detected in voles was as much as three times higher (2.23%) than that in the mice of genus *Apodemus* (0.79%), though the difference was not significant (chi-square = 2.10, *p* = 0.15, Cohen’s w = 0.154, small effect size). *Sarcocystis* spp. were found in rodents collected in 6 out of the 14 localities 42.86% (95% CI = 17.66–68.42%). Parasites were determined in the northern, central, and eastern parts of Lithuania (Figure 1). The highest detection rates were established in Užpaliai (eastern Lithuania) with 5.32% and in Aukštikalniai (northern Lithuania) with 3.77%. The number of individuals tested in the localities where *Sarcocystis* were not detected ranged from 3 to 35 (in six localities) and from 89 to 105 in the two remaining localities.

### 3.2. Molecular Characterization of Sarcocystis *spp.* in Small Mammals

Amplification products were seen only after the second step of nested PCR. The amplification of four genetic loci was successful with positive controls. However, the molecular analysis of the analyzed samples was successful only when using primers that amplified *28S* rDNA and *cox1* products. The amplification and sequencing of *18S* rDNA resulted in unspecific microorganisms and coccidia. While only unspecific bands, which were smaller than expected, were obtained with the primers targeting *ITS1*.

Overall, nine *Sarcocystis* spp. isolates were successfully characterized within partial *cox1* and *28S* rDNA. Based on the comparison of the obtained 619 bp long *cox1* and 726–735 bp long *28S* rDNA sequences, four *Sarcocystis* species (*S*. *myodes*, *Sarcocystis* cf. *strixi*, *Sarcocystis* sp. Rod1, and *Sarcocystis* sp. Rod2) were identified (Table 4). In particular, in this work, *S*. *myodes*—as previously described in *C*. *glareolus* [32]—was found in four rodent species: *A*. *agrarius*, *A*. *flavicollis*, *C*. *glareolus*, and *M*. *arvalis*. *Sarcocystis* cf. *strixi* was identified in a single sample of *A*. *flavicollis*. *Sarcocystis* sp. Rod1 was confirmed in *M*. *arvalis* and *M*. *oeconomus*, and *Sarcocystis* sp. Rod2 was detected in two pooled samples of *M. arvalis*.

Two of the identified species, *S*. *myodes* and *Sarcocystis* sp. Rod1, had the highest genetic similarity with each other, as well as with the *S*. *ratti* from the black rat (*Ratus rattus*) [32,41]. At the *cox1* gene, the sequences of *S. myodes* and *Sarcocystis* sp. Rod1 exhibited a difference of only 0.32%. In the case of the *28S* rDNA gene, the sequences obtained from *S. myodes* in this study shared an identity ranging from 99.18% to 100%, as well as displayed a similarity of 97.28% to 97.82% when compared to the two sequences of *Sarcocystis* sp. Rod1. The two *28S* rDNA sequences of *Sarcocystis* sp. Rod1 showed a difference of 0.27%. Regarding the *cox1* gene, the sequences of *Sarcocystis* cf. *strixi* exhibited a 100% identity to *S. strixi*, which was isolated from the intestinal mucosal scraping of the barred owl (*Strix varia*) [71]. Additionally, they shared a 99.52% similarity with the *S*. *lutrae* obtained from predatory mammals [72] and the *S*. *lari* obtained from the birds of the family Laridae [73]. In contrast, the *28S* rDNA sequences of *Sarcocystis* cf. *strixi* exhibited a similarity of 98.91% to *S. strixi* and less than 96% when compared to other *Sarcocystis* spp. Additionally, when analyzing the *cox1* region, *Sarcocystis* sp. Rod2 could not be distinguished from several examples of *Sarcocystis* spp. that use birds as intermediate hosts. However, based on *28S* rDNA, the sequences of *Sarcocystis* sp. Rod2 showed a similarity of up to 97.25% to the *Sarcocystis* spp. that utilize birds and predatory mammals (Carnivora) as their intermediate hosts [29].

**Figure 2:**
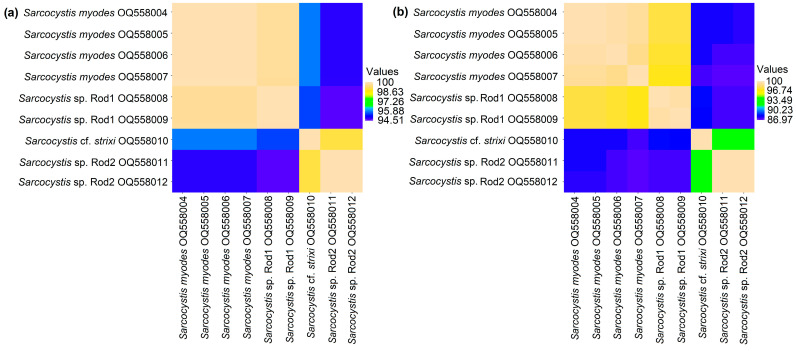
The genetic comparison between the *Sarcocystis* isolates obtained in this work was on the basis of the *cox1* (**a**) and *28S* rRNA (**b**) sequences. The GenBank accession numbers are shown next to the species names.

The genetic comparison of nine *cox1* sequences obtained in this study revealed the presence of four haplotypes, which corresponded to four identified *Sarcocystis* species (Figure 2a). In terms of the *cox1* gene, the genetic differences between *S. myodes* and *Sarcocystis* sp. Rod1, as well as between *Sarcocystis* cf. *strixi* and *Sarcocystis* sp. Rod2, did not exceed 1%. On the other hand, the *28S* rDNA gene exhibited higher interspecies variability compared to *cox1* (Figure 2b). A total of seven *28S* rDNA haplotypes were identified and, based on *28S* rDNA, the differences between the four *Sarcocystis* species exceeded 2%, with intraspecific genetic variabilities of up to 0.8%.

### 3.3. Phylogenetic Relationships between Identified Sarcocystis Species

**Figure 3:**
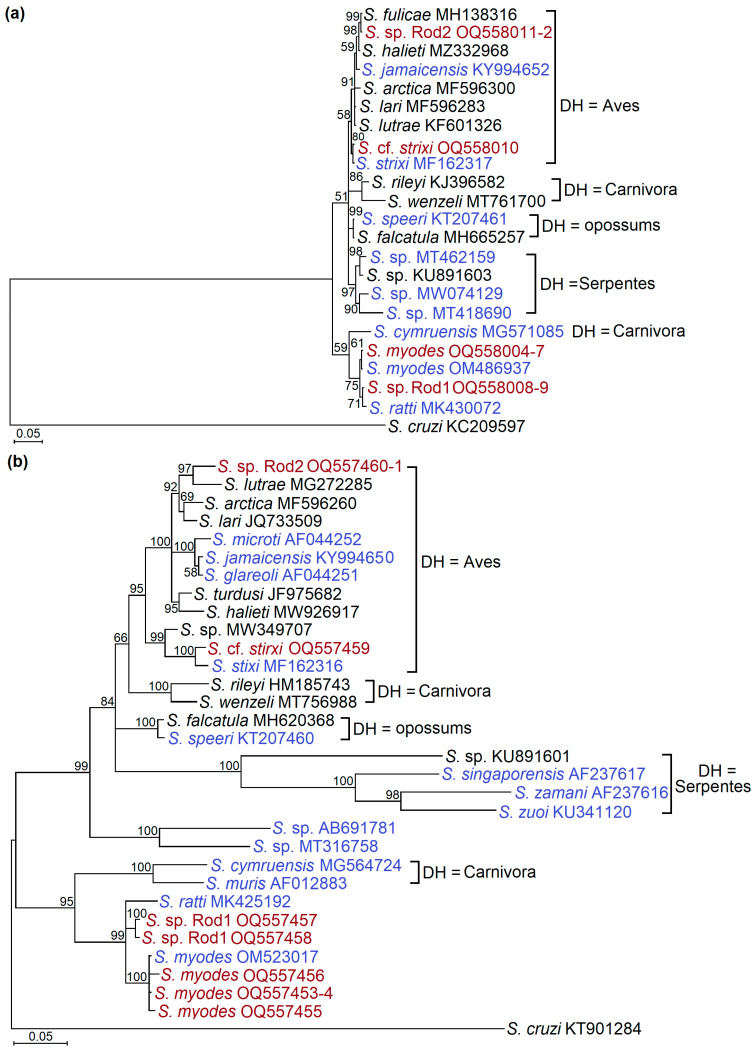
The phylogenetic analysis of the selected *Sarcocystis* spp. based on *cox1* (**a**) and *28S* rDNA (**b**) sequences. Trees were generated using the Bayesian method, which was scaled according to the branch length and rooted on *S*. *cruzi*. Sequences generated in this work are presented in dark red, while the *Sarcocystis* spp. that uses rodents as their intermediate hosts are displayed in indigo. The bootstrap support values are indicated next to the branches. DH = definitive hosts.

Significantly higher bootstrap support values were obtained in the phylogenetic tree that was obtained using *28S* rDNA sequences (Figure 3a) than those obtained in the tree constructed from *cox1* sequences (Figure 3b). Based on both loci, four of the *Sarcocystis* species distinguished in the current work were remote from *Sarcocystis* spp. and were characterized by a rodent–snake life cycle. In general, *Sarcocystis* cf. *strixi* and *Sarcocystis* sp. Rod2 were most closely related with *Sarcocystis* spp., which use birds as their definitive hosts, while *S*. *myodes* and *Sarcocystis* sp. Rod1 were grouped together with *Sarcocystis* spp., which employ predatory mammals as their definitive hosts. In the *28S* rDNA phylogram, the isolates of *S*. *myodes* composed a common cluster. *Sarcocystis* cf. *strixi* was grouped with the *S*. *strixi* from the barred owl (*Strix varia*) [71], and it was most closely related with the *Sarcocystis* sp. (MW349707) isolated from the intestinal mucosa of the boreal Tengmalm’s owl (*Aegolius funereus*) [74]. *Sarcocystis* sp. Rod1 was placed into one cluster together with the *S*. *myodes* and *S*. *ratti* described in the rodents from the Baltic States [32,41], and *Sarcocystis* sp. Rod2 was a sister taxon to the *S*. *lutrae* detected in various predatory mammals [72]. It is noteworthy that, on the basis of *cox1*, *Sarcocystis* sp. Rod1 was found to be more closely related to *S*. *ratti* than to *S*. *myodes*.

## 4. Discussion

### 4.1. Evaluation of the Sarcocystis *spp.* Prevalence in Different Species of Small Mammals

By means of a molecular analysis, *Sarcocystis* spp. were detected in the skeletal muscles of two *Apodemus* species and three vole species of genus *Clethrionomys* and *Microtus* (Table 3) from orchards and berry plantations in Lithuania. The parasites analyzed were not found in the five individuals of the insectivorous mammals from the genus *Sorex* that belong to the order Eulipotyphla. The overall prevalence of *Sarcocystis* spp. was low, reaching 1.38%. Relatively higher, however, a not significant infection rate of *Sarcocystis* spp. was established in voles (2.23%) than in the mice of the genus *Apodemus* (0.79%).

The prevalence of *Sarcocystis* was not related to the abundance of small mammal species tested. The most numerous species were *M. arvalis* (28.7%), *A. flavicollis* (27.9%), *A. agrarius* (22.2%), and *C. glareolus* (12.0%) with respect to all of the trapped small mammals [21]—this being not in line with their infection rate (Table 3). Five of the sites where the infection was registered are age-old apple orchards (i.e., sites Aukštikalniai, Ažuožeriai, Tytuvėnai, Dembava, and Luksnėnai), and one site, Užpaliai, is a young raspberry plantation.

There is a lack of research on the prevalence of *Sarcocystis* spp. in small mammals worldwide [75]. Researchers have suggested that the infection rates of various *Sarcocystis* depend on the parasite species, intermediate host species, geographic area, as well as on the availability and abundance of definitive hosts in the area under study [32,50,75]. Previous studies conducted in Lithuania showed the tendency for *Sarcocystis* spp. infection rates to differ depending on the species of small mammals [53,54,55]. In two species of the genus *Apodemus*, *A*. *agrarius* and *A*. *flavicollis*, the prevalence of *Sarcocystis* spp. reached 1.18% [53,54]. Thus, the occurrence rate of the examined parasites in the mice of the genus *Apodemus* (Table 3) is in congruence with the previous studies carried out in Lithuania. The prevalence of *Sarcocystis* spp. in the three vole species most comprehensively examined in the country (*C. glareolus*, *M*. *agrestis*, and *M. arvalis*) ranged from 1.81 to 5.26% in the environs of Lake Drūkšiai [55], to 11.40 to 20% in the Kamasta landscape reserve [53]. Based on the data of the previous investigations conducted in Lithuania and the current study, the infection rates of the *Sarcocystis* spp. in the muscles of small mammals mainly depend on the host species and the environment.

### 4.2. Sarcocystis Species Identification and Richness in Small Mammals Inhabiting Orchards

The sequence comparison of *cox1* and *28S* rDNA indicated the presence of four *Sarcocystis* species (*S*. *myodes*, *Sarcocystis* cf. *strixi*, *Sarcocystis* sp. Rod1, and *Sarcocystis* sp. Rod2) in the small mammals that were collected in the orchards of Lithuania (Figure 3, Table 4). *Sarcocystis myodes* was originally described in the skeletal muscles of *C*. *glareolus* [32]; meanwhile, in the current work, this species was apart from the already known intermediate hosts found in *A*. *agrarius*, *A*. *flavicollis*, and *M*. *arvalis*. Thus, this *Sarcocystis* species is not strictly host-specific and could infect the mammals belonging to the families Cricetidae (*C*. *glareolus*, *M*. *arvalis*) and Muridae (*A*. *agrarius*, *A*. *flavicollis*). The intraspecific variation of *S*. *myodes* amounted to 0.82% within the *28S* rDNA fragment analyzed. Based on *28S* rDNA, *S*. *myodes* displayed a great genetic similarity to *Sarcocystis* sp. Rod1 (Figure 2 and Table 4), which was identified in two vole species—*M*. *arvalis* and *M*. *oeconomus*. Future research on the morphological and genetic characterization of *Sarcocystis* sp. Rod1, as well as on the determination of the spectrum of intermediate hosts, are needed.

Additionally, the results of the current study showed that one isolate from *A*. *flavicollis* was 100% identical to *S*. *strixi* within a 619 bp fragment of *cox1*. It also showed a 98.91% similarity with *S*. *strixi* (Table 4) (whose gamma gene knockout mice is an experimental intermediate host, and the barred owl is a definitive host [71]). In the previous study, *18S* rDNA, *28S* rRNA, and *cox1* loci were used for the genetic characterization of *S*. *strixi* [71]. This *Sarcocystis* species was described in the USA. On the basis of the present work, it is very likely that *A*. *flavicollis* might be a natural intermediate host of *S*. *strixi* in Europe; however, further comprehensive investigations of *Sarcocystis* cf. *strixi* from the *A*. *flavicollis* on sarcocysts morphology, as well as the genetic identification in complete or nearly complete *18S* rDNA, *28S* rRNA, and *cox1*, are required. Furthermore, *Sarcocystis* sp. Rod2 were identified in the two isolates of *M*. *arvalis* and showed the greatest genetic similarity to the several *Sarcocystis* spp. (such as *S*. *arctica*, *S*. *calchasi*, *S*. *columbae*, *S*. *cornixi*, *S*. *corvusi*, *S*. *fulicae*, *S*. *halieti*, *S*. *lari*, *S*. *lutrae*, and *S*. *turdusi*) that use birds and predatory mammals as their intermediate hosts, as well as predatory or omnivorous birds as their definitive hosts [76,77,78,79,80,81,82]. Interestingly, the *S*. *tupaia* from small mammals—namely, from tree shrews (*Tupaia belangeri chinensis*)—also demonstrated the closest similarity within *18S* rDNA to the various *Sarcocystis* species that are distinguished by a bird–bird life cycle [49].

The studies on *Sarcocystis* spp. in the genus *Apodemus* are very scarce, and only two species, *S*. *microti* and *S*. *sebeki*, have been described in these hosts [28,29,83]; this contrasts with the more than dozen *Sarcocystis* spp. detected in voles [32,39]. Previous investigations of *Sarcocystis* spp. in the voles and mice of the genus *Apodemus* relied mainly on morphological and life cycle studies [29], and only *S*. *clethrionomyelaphis*, *S*. *glareoli*, *S*. *microti*, and *S*. *myodes* have been examined by means of DNA sequence analysis [32,34,36,37,38]. Therefore, it is difficult to compare the species identified in this work with those previously described in the same or taxonomically closely related hosts. Our further research should be directed toward the isolation of individual sarcocysts from the muscles of small mammals. In addition, their characterization will be achieved via light and electron microscopy, as well as by DNA sequence analysis, at several loci.

It is noteworthy that, in the present study, only two species were reliably distinguished by an analysis of the partial *cox1* sequences, while two species were identified using *28S* rDNA (Figure 3, Table 4). When investigating the *Sarcocystis* spp. from small mammals, other previous studies have also indicated higher interspecific variability within *28S* rDNA when compared to *cox1* [30,32,41,75]. Apart from *28S* rDNA and *cox1*, various genetic markers have been applied for the genetic identification of the *Sarcocystis* spp. in small mammals. Most of these species are characterized by *18S* rDNA, *28S* rDNA, and *cox1* [41]. The first investigations of the *ITS1* region in the *Sarcocystis* spp. from small mammals did not reveal significant BLAST similarity hits [30,41]. However, as the *ITS1* sequence database accumulated, further examinations showed that this highly variable region could be very useful in differentiating the closely related *Sarcocystis* spp. from small mammals [32,50]. It has also been shown recently that a complete *ITS1*–*5.8S* rDNA–*ITS2* region could be useful for the evolutionary studies of *Sarcocystis* spp. from small mammals [47]. Other investigators demonstrated that mitochondrial cytochrome b (*cytb*) was a better choice than *18S* rDNA and *cox1* for the discrimination of the closely related *S*. *cymruensis* and *S*. *ratti* that parasitize rats [35]. In addition to the genetic loci discussed, *S*. *attenuati* was characterized at two apicoplast genes—RNA polymerase beta subunit (*rpoB*) and caseinolytic protease C (*clpC*) [50]. The primary results indicated that these two apicoplast DNA loci can be potentially valuable for the discrimination of *Sarcocystis* spp. from small mammals. Considering the existing genetic studies on *Sarcocystis* spp. in small mammals, it is recommended that the *Sarcocystis* species identified in this study be further characterized in the future via more informative genetic markers. This would help in obtaining a more comprehensive understanding of their genetic profiles.

Small mammals can adapt to any terrestrial environment, including areas closely related to the human environment [84]. To the best of our knowledge, research on the extent of *Sarcocystis* spp. richness exclusively in orchards has not yet been conducted. The present study showed the presence of four *Sarcocystis* spp. in the muscle tissues of small mammals inhabiting orchards. Of these species, *Sarcocystis* sp. Rod1 and *Sarcocystis* sp. Rod2 are potentially new species. The possible pathogenicity of genetically determined *Sarcocystis* species should be further examined as small mammals have an important role in the epidemiology of numerous parasitic diseases [75].

### 4.3. Ecological and Phylogenetic Insights on the Definitive Hosts of Detected Sarcocystis Species

A coevolution of *Sarcocystis* spp. from small mammals to their definitive hosts, rather than to their intermediate hosts, have been shown in a series of studies [61,85]. Currently, possible definitive hosts of *Sarcocystis* species are suggested based on phylogenetic results [86,87,88,89]. The phylogenetic analysis of this work showed that the presumed definitive hosts of *S*. *myodes* and *Sarcocystis* sp. Rod1 are predatory mammals, while the assumed definitive hosts of *Sarcocystis* cf. *strixi* and *Sarcocystis* sp. Rod2 are birds of prey (Figure 3). Based on *28S* rDNA, two main clades were defined in the phylogenetic group of *Sarcocystis* spp., whose identified or supposed definitive hosts are birds (Figure 3b). The second lesser species-numerous clades contained *S*. *strixi* (which employs the bared owl as a definitive host), the *Sarcocystis* cf. *strixi* from *A*. *flavicollis*, and the *Sarcocystis* sp. (MF162316) from the intestinal mucosa of the Tengmalm’s owl (*Aegolius funereus*) [71,74]. Thus, the definitive hosts of these *Sarcocystis* spp. are members of the order Strigiformes, whereas representatives of the genus *Accipiter*, *Buteo*, and *Haliaeetus* belong to the order Accipitriformes, which were identified as the definitive hosts of species-numerous phylogenetic clades by means of laboratory experiments or DNA analysis [36,37,76,79,80,81,82]. In view of what is stated above, the birds of prey of the order Accipitriformes are presumed to be the definitive hosts of *Sarcocystis* sp. Rod1.

The current study showed no evidence of the existence of the *Sarcocystis* species being transmitted by snakes in Lithuanian orchards. By contrast, a recent molecular study conducted in the peri-urban area in northeast Spain suggested at least three *Sarcocystis* spp., with a life cycle of rodents as intermediates hosts and snakes as definitive hosts [74]. Although the adder (*Vipera berus*) and grass snake (*Natrix natrix*) are not uncommon in Lithuania, with the grass snake being frequently encountered near human settlements, these snake species have not yet been observed in commercial orchards to date [90].

## 5. Conclusions

Based on the pooling of muscle samples, pepsin digestion, the nested PGR targeting of *cox1* and *28S* rRNA, and sequencing, a low *Sarcocystis* spp. prevalence (1.38%, 95% CI = 0.68–2.52) was determined in the small mammals that were collected from commercial orchards and berry plantations in Lithuania. According to the current knowledge, the infection rates of *Sarcocystis* spp. in small mammals are mostly dependent on the host species and environment.

Four *Sarcocystis* spp., *S*. *myodes*, *Sarcocystis* cf. *strixi*, *Sarcocystis* sp. Rod1, and *Sarcocystis* sp. Rod2, were identified in the present study. Three new intermediate hosts (*A*. *agrarius*, *A*. *flavicollis*, and *M. arvalis*) were confirmed for the recently described *S*. *myodes*. Molecular results suggest that *A*. *flavicollis* might be a natural intermediate host of *S*. *strixi* in Europe, and that *Sarcocystis* sp. Rod1 and *Sarcocystis* sp. Rod2 are potentially a new species. Phylogenetic analysis showed that mammals and birds are most likely the definitive hosts of *S*. *myodes* and *Sarcocystis* sp. Rod1, and *Sarcocystis* cf. *strixi* and *Sarcocystis* sp. Rod2, respectively. Additional genetic characterization that uses more genetic markers is required to further understand the detected *Sarcocystis* species. Moreover, a comprehensive morphological characterization of the *Sarcocystis* species discovered in this study should be carried out with light and electron microscopy. Additionally, it is crucial to investigate the definitive hosts and ascertain the potential pathogenicity of the identified parasites.

## Figures and Tables

**Table 1:** The number of the examined species and individuals collected in the 14 sites. The number of pooled groups are presented in parenthesis.

Sample Site	Host Species							
	*Apodemus agrarius*	*Apodemus flavicollis*	*C* *lethrionomys glareolus*	*Microtus agrestis*	*M* *icrotus arvalis*	*M* *icrotus oeconomus*	*S* *orex araneus*	*S* *orex minutus*
Aukštikalniai	11 (2)	4 (1)			36 (5)	2 (1)		
Naradava	42 (5)	36 (5)		5 (1)	6 (1)			
Mieliūnai					16 (2)			
Užpaliai	24 (3)	3 (1)			69 (7)		2 (1)	
Kalpokai					3 (1)			
Ažuožeriai	7 (1)	29 (3)			40 (5)	2 (1)		
Tytuvėnai		28 (3)	28 (3)	5 (1)				
Taujėnai		7 (1)			6 (1)			
Dembava		17 (2)						
Barčiai	10 (1)	9 (1)	8 (1)		8 (1)			
Luksnėnai	7 (2)	42 (6)	22 (2)		10 (2)			3 (1)
Gaurė					5 (1)			
Šešuolėliai		13 (2)	9 (1)					
Žiežmariai	45 (5)	54 (6)	6 (1)					

**Table 2:** Oligonucleotide primers used for the nested PCR targeting: *18S* rDNA, *28S* rDNA, *ITS1*, and *cox1*.

Primer Name	Sequence	Region	Round of Nested PCR	T_a_, °C	Approximate Length of PCR Product ^a^
Sgrau181 ^PS^	AAGTATAAGCTTTTATACGGCGAAA	*18S* rDNA	First	61	900
Sgrau182 ^PS^	TCGCAGTAGTTCGTCTTTAACAAA				
Sgrau183 ^PS^	TGGATAACCGTGGTAATTCTATG		Second	59	750
Sgrau184 ^PS^	TCCCTATTAATCATTACTTCAGTCCTA				
Sgrau281 ^PS^	GCGGAGGAAAAGAAAATAACAAT	*28S* rDNA	First	61	900
Sgrau282 ^PS^	CTATCGCTTAGGACCGGCTA				
Sgrau283 ^PS^	GTGAACAGGGAAGAGCTCAA		Second	59	800
Sgrau284 ^PS^	CTCCACGTCTTCCTACTCATTG				
SU1F ^b^	GATTGAGTGTTCCGGTGAATTATT	*ITS1* ^d^	First	59	1100
5.8SR2 ^b^	AAGGTGCCATTTGCGTTCAGAA				
SgrauITS3 ^PS^	GGGAAGTTTTGTGAACCTTAACACT		Second	57	950
SgrauITS4 ^PS^	ATTCTGCAATTCACATTGCGTTT				
SF1 ^c^	ATGGCGTACAACAATCATAAAGAA	*cox1*	First	59	1100
SR5 ^c^	TAGGTATCATGTAACGCAATATCCAT				
SgraucoF1 ^PS^	GGTTTTGGTAACTACTTTGTACCG		Second	59	660
SgraucoR1 ^PS^	ACCTCTAATCCTACGGTCATCA				

^Ta^ the primer annealing temperatures used for PCR, ^a^ the length of the product, which varies depending on the *Sarcocystis* species. A comparison of the *Sarcocystis* species’ high variation in the length of loci was previously observed in *ITS1* and in some domains of *18S* rDNA and *28S* rDNA [42,60,61]. ^PS^ the present study, ^b^ [62], ^c^ [61], ^d^ the region that contains complete *ITS1*, as well as short fragments of *18S* rDNA and *5.8S* rDNA.

**Table 3:** The detection rates of *Sarcocystis* spp. in the examined species of small mammals and in the analyzed localities. The prevalence from the pooled samples were calculated according to B.J. Biggerstaff and G. Hepworth [66,67,68], and by using the Excel program as presented in [67].

Sample	Number of Individuals Screened	Number of Pools Analyzed	Number of Positive Pools	Prevalence (95% Confidence Intervals)
Species				
*Apodemus agrarius*	146	19	1	0.68 (0.04–3.26)
*Apodemus flavicollis*	242	31	2	0.84 (0.15–2.75)
Mice	388	50	3	0.79 (0.21–2.12)
*Clethrionomys glareolus*	73	9	1	1.34 (0.08–6.43)
*Microtus agrestis*	10	2	0	0
*Microtus arvalis*	199	26	4	2.16 (0.71–5.18)
*Microtus oeconomus*	4	2	1	24.87 (1.64–81.95)
Voles	292	39	6	2.23 (0.92–4.59)
*Sorex araneus*	2	1	0	0
*Sorex minutus*	3	1	0	0
Shrews	5	2	0	0
Total	679	91	9	1.38 (0.68–2.52)
Sites				
1	53	9	2	3.77 (0.73–11.86)
2	78	10	1	1.27 (0.08–6.09)
3	35	4	0	0
4	17	2	1	5.32 (0.39–31.68)
5	5	1	0	0
6	3	1	0	0
7	84	14	1	1.18 (0.07–5.67)
8	16	2	0	0
9	89	12	0	0
10	22	3	0	0
11	13	2	0	0
12	61	7	1	1.65 (0.10–8.11)
13	98	12	3	3.49 (0.94–9.57)
14	105	12	0	0

**Table 4:** Identification and genetic variability of the *Sarcocystis* spp. isolated from rodents collected in Lithuania.

Feature	*Sarcocystis* Species			
	*S*. *myodes*	*Sarcocystis* cf. *strixi*	*Sarcocystis* sp. Rod1	*Sarcocystis* sp. Rod2 *
IH	*Apodemus agrarius, Apodemus flavicollis, Clethrionomys glareolus,* *Microtus arvalis*	*Apodemus flavicollis*	*Microtus arvalis*, *Microtus oeconomus*	*Microtus arvalis*
	Sequence similarity			
*Cox1*	100% *S*. *myodes*, 99.68% *Sarcocystis* sp. Rod1, 99.19% *S*. *ratti*, 95.80% *S*. *strixi*	100% *S*. *strixi*, 99.52% *S*. *lutrae*, 99.52% *S*. *lari*	99.68% *S*. *myodes*, 99.52% *S*. *ratti*, 95.48% *S*. *strixi*	100% *S*. *fulicae*, 100% *S*. *cornixi*, 99.82% *S*. *columbae*, 99.82% *S*. *corvusi*, 99.82% *S*. *turdusi*, 99.82% *S*. *halieti*
*28S* rDNA	99.18–100% *S*. *myodes*, 97.28–97.82% *Sarcocystis* sp. Rod1, 95.92–96.46% *S*. *ratti*, 88.36–88.90% *S*. *cymruensis*, 88.35–88.89% *S*. *muris*	98.91% *S*. *strixi*, 95.37% *Sarcocystis* sp. (MW349707), 95.24% *S*. *lari*, 94.97% *S*. *turdusi*	97.28–97.82% *S*. *myodes*, 97.28–97.55% *S*. *ratti*, 90.24–90.26% *S*. *cymruensis*, 89.17% *S*. *muris*	97.11–97.25% *S*. *arctica*, 97.12% *S*. *lari*, 97.12% *S*. *lutrae*

* *Sarcocystis* sp. Rod2 was identified in two pooled samples of the same host species, *M. arvalis*, whereas the other *Sarcocystis* spp. were detected in a single pooled sample of the certain host species.

## Data Availability

The *28S* rDNA and *cox1* sequences of *Sarcocystis* spp. obtained in the present study were submitted to the GenBank database under accession numbers OQ557453-OQ557461 and OQ558004-OQ558012, respectively.
